# Spermidine as a promising anticancer agent: Recent advances and newer insights on its molecular mechanisms

**DOI:** 10.3389/fchem.2023.1164477

**Published:** 2023-04-06

**Authors:** Parteek Prasher, Mousmee Sharma, Sachin Kumar Singh, Monica Gulati, Dinesh Kumar Chellappan, Rashi Rajput, Gaurav Gupta, Alibek Ydyrys, Marzhan Kulbayeva, Ahmad Faizal Abdull Razis, Babagana Modu, Javad Sharifi-Rad, Kamal Dua

**Affiliations:** ^1^ Department of Chemistry, University of Petroleum and Energy Studies, Dehradun, India; ^2^ Department of Chemistry, Uttaranchal University, Dehradun, India; ^3^ School of Pharmaceutical Science, Lovely Professional University, Phagwara, India; ^4^ Faculty of Health, Australian Research Centre in Complementary and Integrative Medicine, University of Technology Sydney, Ultimo, NSW, Australia; ^5^ School of Pharmacy, International Medical University, Bukit Jalil, Malaysia; ^6^ Discipline of Pharmacy, Graduate School of Health, University of Technology Sydney, Ultimo, NSW, Australia; ^7^ School of Pharmacy, Suresh Gyan Vihar University, Jaipur, Rajasthan, India; ^8^ Department of Pharmacology, Saveetha Dental College, Saveetha Institute of Medical and Technical Sciences, Saveetha University, Chennai, India; ^9^ Biomedical Research Centre, Al-Farabi Kazakh National University, Almaty, Kazakhstan; ^10^ Department of Biophysics, Biomedicine and Neuroscience, Al-Farabi Kazakh National University, Almaty, Kazakhstan; ^11^ Department of Food Science, Faculty of Food Science and Technology, Universiti Putra Malaysia, Selangor, Malaysia; ^12^ Natural Medicines and Products Research Laboratory, Institute of Bioscience, Universiti Putra Malaysia, Selangor, Malaysia; ^13^ Department of Biochemistry, Faculty of Science, University of Maiduguri, Maiduguri, Nigeria; ^14^ Facultad de Medicina, Universidad del Azuay, Cuenca, Ecuador

**Keywords:** spermidine, polyamines, anticancer immunosurveillance, diagnostic marker, cell proliferation, anticancer properties

## Abstract

Spermidine is a naturally occurring polyamine compound found in semen. It is also found in several plant sources and boasts a remarkable biological profile, particularly with regards to its anticancer properties. Spermidine specifically interferes with the tumour cell cycle, resulting in the inhibition of tumor cell proliferation and suppression of tumor growth. Moreover, it also triggers autophagy by regulating key oncologic pathways. The increased intake of polyamines, such as spermidine, can suppress oncogenesis and slow the growth of tumors due to its role in anticancer immunosurveillance and regulation of polyamine metabolism. Spermidine/spermine N-1-acetyltransferase (SSAT) plays a critical role in polyamine homeostasis and serves as a diagnostic marker in human cancers. Chemically modified derivatives of spermidine hold great potential for prognostic, diagnostic, and therapeutic applications against various malignancies. This review discusses in detail the recent findings that support the anticancer mechanisms of spermidine and its molecular physiology.

## Introduction

Spermidine is a natural biomolecule which has been reported to possess a broad spectrum of health improving effects, that includes remarkable anti-inflammatory effects (Madeo et al., 2018b; Liu et al., 2020). It is also a potent antioxidant, and reportedly improves the respiratory function (Madeo et al., 2018a; Kim et al., 2021). Spermidine possesses a positive charge at the physiological pH that enables its interaction with oppositely charged DNA and RNA (Madeo et al., 2018a). Dietary intake of spermidine reduces the risk of neurodegeneration, metabolic diseases, heart ailments, and cancer. Furthermore, spermidine-induced autophagy slows the rate of cognitive decline due to its ability to clear amyloid-beta plaques in the brain (Madeo et al., 2018b). Spermidine supplementation also enhances mitochondrial metabolism, and translational activity. The anticancer properties of spermidine are of particular interest as it is known to reduce the cancer-related mortality in humans (Pietrocola et al., 2019). The association between tumorigenesis and spermidine is determined by the regulation of polyamine metabolism, surveillance of immune system, apoptosis, and autophagy (Nampoothiri et al., 2023). Higher uptake of polyamines is known to suppress the tumor progression. Spermidine exhibits cell-autonomous effects on cancer cells, in addition to impacting their discourse with the immune effectors that result in expediting the identification of tumor-associated antigens and eventually cancer cell death (Fan et al., 2020). Spermidine-mediated autophagy inhibits activation of apoptosis in cancer cells (Madeo et al., 2019) and is associated with tissue development and cell differentiation (Ni and Liu, 2021).

## Spermidine-induced autophagy

**FIGURE 1:**
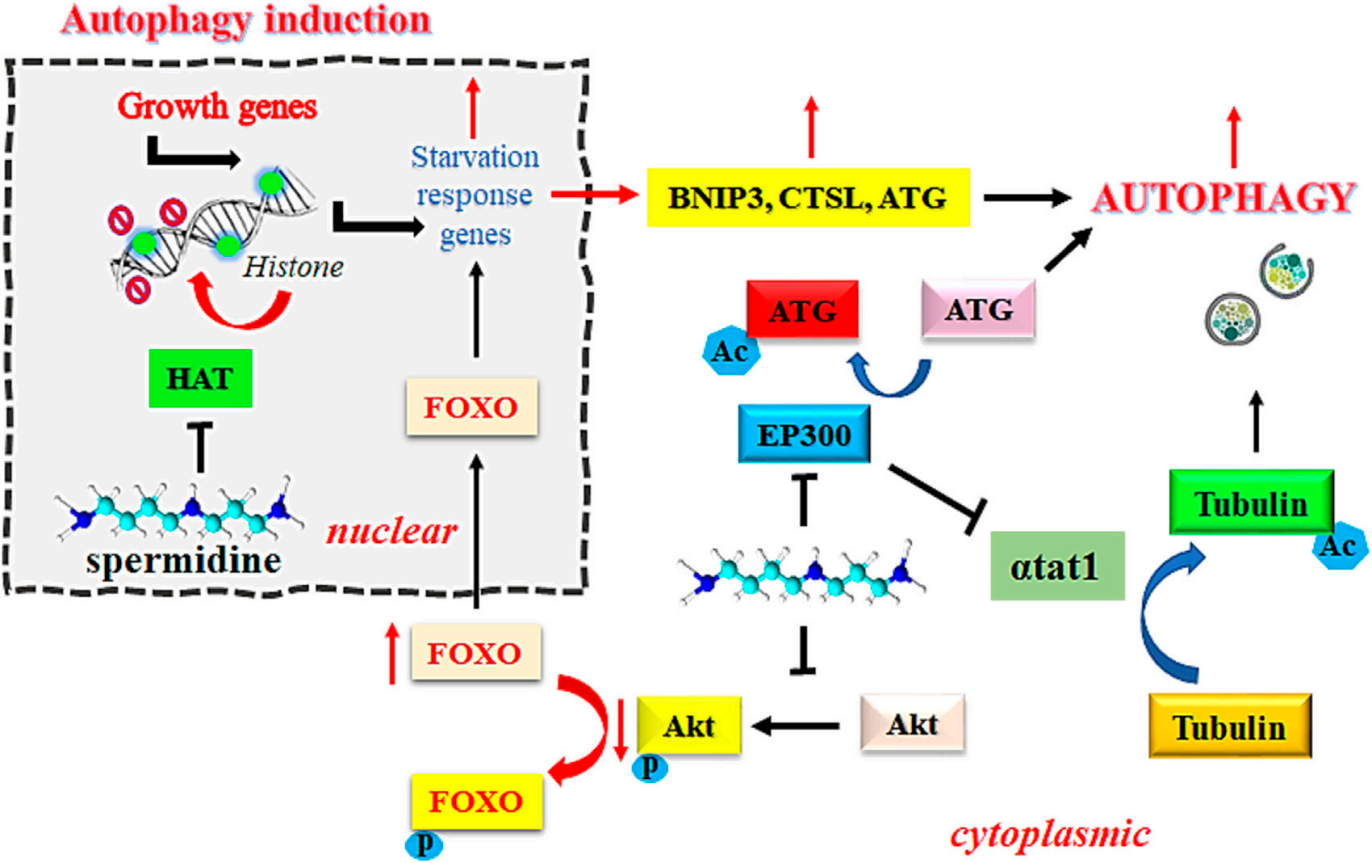
Mechanism of spermidine-induced autophagy. Abbreviations: FOXO: Fork-head transcription factors of the O class; Akt, Ak strain transforming; BNIP3, BCL2/adenovirus E1B 19 kDa protein-interacting protein 3; Ac, acylated; P, phosphorylated; HAT, histone acetyltransferase; EP300, E1A-associated protein p300; αtat1, α-tubulin acetyltransferase; CTSL, cathepsin L; ATG, autophagy related gene.

EP300 serves as a sensor for nutrient-dependent acetyl-CoA that is known to inhibit autophagy related proteins, including ATG5, ATG7, ATG12, BECN1, and LC3. However, the inhibition of EP300 by spermidine induces autophagic flux in mammalian cells (Sacitharan et al., 2018). Intracellular accumulation of toxic debris in morbid cells is directly associated with the pathogenesis of cancer. Removal of such debris by autophagy is an established mechanism to prevent the onset of oncogenesis (Yun and Lee, 2018). Spermidine functions as an endogenous inhibitor of acetyl transferase EP300 due to its steric competition with Acetyl CoA for binding to the enzyme catalytic site. Inhibition of acetyltransferase EP300 by spermidine is known to induce autophagy, which is one of the desirable approaches in the treatment of cancer. Spermidine reportedly inhibits the tendency of recombinant human EP300 protein to acetylate its natural substrate histone H3 at physiological concentration of acetyl-CoA (acetyl donor) at 10 µM. This effect was further improved on increasing the acetyl-CoA level to 100 μM, thereby suggesting a competitive inhibition by spermidine (Pietrocola et al., 2015). Figure 1 explains the mechanism of induction of autophagy by spermidine.

Pietrocola et al. (2019) also reported that spermidine induces autophagy in cancer cells to improve the anticancer immunosurveillance. This has been proved therapeutically beneficial for reducing the risk associated with hepatocellular carcinoma, and colorectal cancer, in addition to ameliorating the adaptive branch of immune system (Pietrocola et al., 2019). *In vivo* experiments have demonstrated the suppression of hepatocellular carcinoma by oral spermidine at 100 µM concentration through the triggering of Microtubule associating protein 1S (MAP1S)-mediated autophagy. MAP1S interacts with the autophagy marker LC3 found in mammals, which connects the components of autophagy with microtubules and mitochondria to affect the degradation of autophagosomes. The intraperitoneal, single dose of injection of spermidine at 50 mg/kg body weight in 1-month old wild-type mice or the MAP1S mice induces autophagy (Yue et al., 2017). The depletion of MAP1S causes autophagy defects that result in heightened redox stress, fibronectin-induced liver fibrosis, and liver sinusoidal dilatation (Li et al., 2016). Increased levels of MAP1S in tumor cells improve the survival of patients suffering from clear renal cell carcinoma, and prostate adenocarcinoma (Xu et al., 2016). Furthermore, the interaction with histone deacetylase4 (HDAC4) improves the acetylation of MAP1S, which in turn activates the autophagy flux. Treatment of cells or wild-type mice with spermidine has shown to improve the stability of MAP1S and autophagy signaling pertaining to the depletion of cytosolic HDAC4. Lifelong oral spermidine administration is reported to extend the lifespan in mice by 25%, as evidenced by genetic investigations. This finding has also confirmed that the effect of orally administered spermidine depends on MAP1S-induced autophagy (Yue et al., 2017).

Spermidine-mediated autophagic activation in cervical cancer, including the onset of apoptosis which leads to growth inhibition has been reported by Chen et al. (2018). The investigations on HeLa cells *via* CCK-8 and flow cytometric assays have clearly indicated that spermidine inhibited the HeLa cell-proliferation by arresting the cell cycle in S phase in a dose dependent manner. Further investigations in the presence of Annexin V-FITC/PI-staining in flow cytometry have suggested the onset of apoptosis in HeLa cells by spermidine, while the Western blot analysis established the induction of autophagy (Chen et al., 2018). Furthermore, annexin V is used to stain phosphatidylserine, which is originally present on the inner side of the plasma membrane and is exposed to the exterior during the rupture of the plasma membrane or in the event of cell death (Razvi et al., 2017). This event serves as a hallmark of apoptosis. The rapid rise in the functioning of annexin V positive cells indicates a decreased cell viability due to the induction of apoptosis.

Further findings from the Western blotting experiments have showed a surge in the levels of LC3 II/LC3 I, Atg5, and Beclin 1 proteins in spermidine administered HeLa cells. LC3 serves as a marker for autophagosome membrane functionality and is associated with the formation of autophagic body, whereas the LC3-I/II conversion indicates the number of autophagosomes generated. Beclin 1 is a specific gene associated with autophagy in mammals and with phospholipid inositol triphosphate-kinase (PI3K) it participates in the formation of autophagosomes (Sun et al., 2009). Similarly, Atg5 is known to regulate autophagy and is involved in the elongation of autophagosomes (Kim et al., 2015). An increase in the levels of these three factors in spermidine-treated HeLa cells clearly indicates the onset of autophagy.

Autophagy activity in macrophages is essential for M2-polarization and its dysregulation may result in a proinflammatory state (Liu et al., 2015; Liu et al., 2021). Spermidine induces mitochondrial reactive oxygen species (mtROS) mediated M2-polarization by producing a surge in the levels of H_2_O_2_ and mitochondrial peroxide in the presence of spermidine. mtROS stimulated AMP-activated protein kinase (AMPK) has previously shown an increase in the mitochondrial function and has also shown an upregulation of hypoxia-inducible factor-1α (HIF-1α) (Liu et al., 2020). These events further increase the expression of anti-inflammatory genes and results in the stimulation of autophagy. *In vitro* and animal studies have suggested that the exposure of macrophages with spermidine was found to improve dextran sulfate sodium (DSS)-induced inflammation due to an increased autophagy. Partial blocking of spermidine-induced autophagy by KC7F2, which is a Hif-1α inhibitor suggests the role of the latter in regulating spermidine-induced autophagy at 20 µM in the culture medium. This evidence indicates that spermidine possibly induces catabolic autophagy (Liu et al., 2020).

### Spermidine-induced apoptosis

**FIGURE 2:**
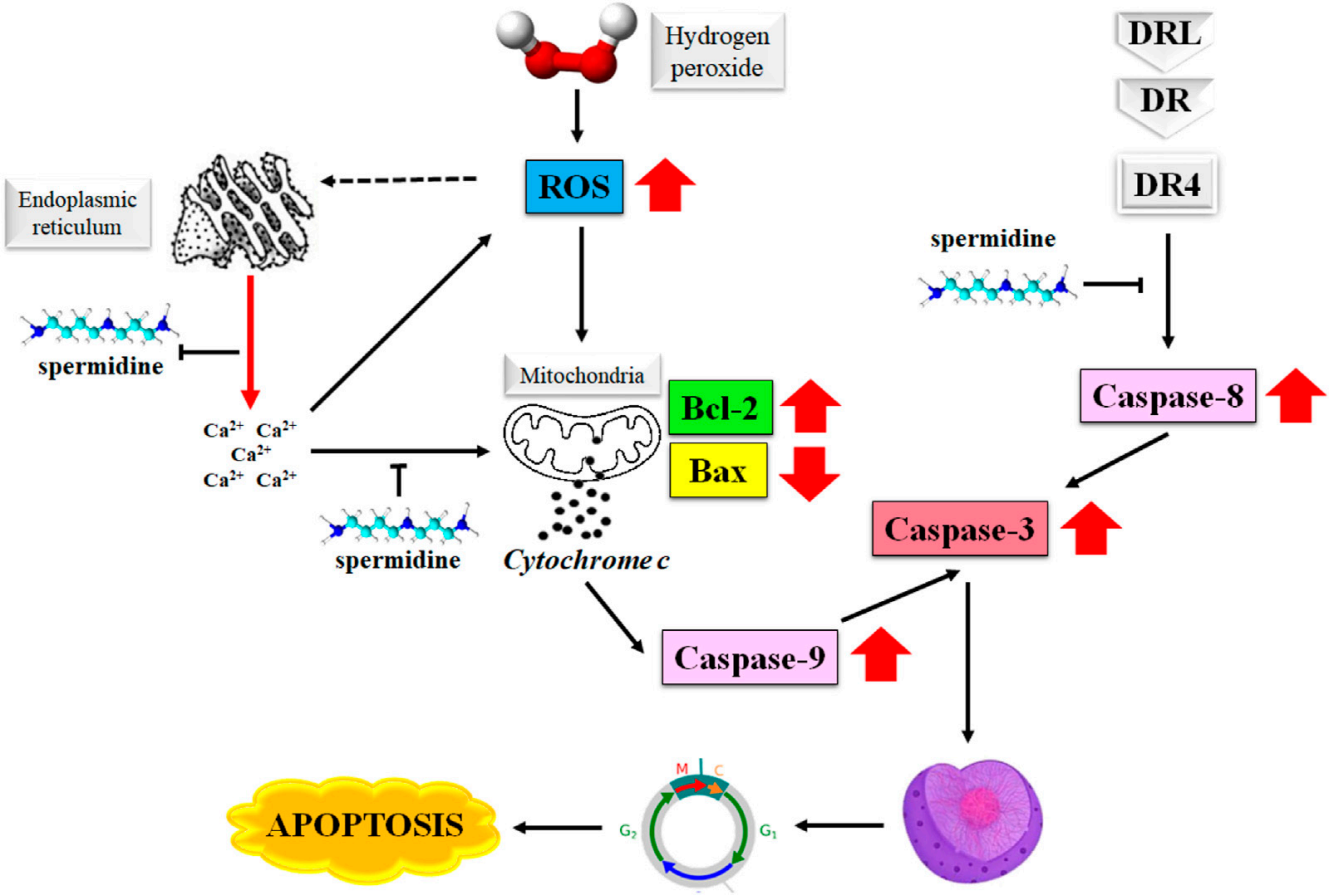
Mechanism of apoptosis induction by spermidine. Abbreviations: ROS, reactive oxygen species; Bax, Bcl-2 associated X protein; DR4, death receptor 4.

The degradation of apoptosis in cancer cells improves their survival as this event increases the time required for the accumulation of mutations that may improve the tumor cell progression and invasiveness. The role played by apoptosis in stimulating angiogenesis, regulation of cell differentiation, and de-regularization of cell proliferation in cancer cells makes it an attractive target in the treatment of cancer (Fulda, 2015; Pistritto et al., 2016; Pfeffer and Singh, 2018). Spermine is known to induce apoptosis in primary human cells as well as the malignant tumor cells by producing a surge in the intracellular level of reactive oxygen species (ROS) (Hwangbo et al., 2023). Figure 2 illustrates the mechanism of the induction of apoptosis by spermidine and its analogues.

Furthermore, the disruption in the mitochondrial membrane potential causes the release of apoptosis-triggering molecules, such as cytochrome c and Smac/DIABLO from the intramembranous space along with a decrease in the Bcl-2 expression. These events eventually determine the extent of spermidine-mediated apoptosis (Gogvadze and Orrenius, 2006). Spermidine is shown to exert antioxidative properties (Rider et al., 2007), however the H_2_O_2_ triggered generation of intracellular ROS is not directly associated with spermidine as evidenced by the suppression of cytosolic Ca^2+^ overload arising from H_2_O_2_-mediated endoplasmic reticulum stress (Kim et al., 2021).

**FIGURE 3:**
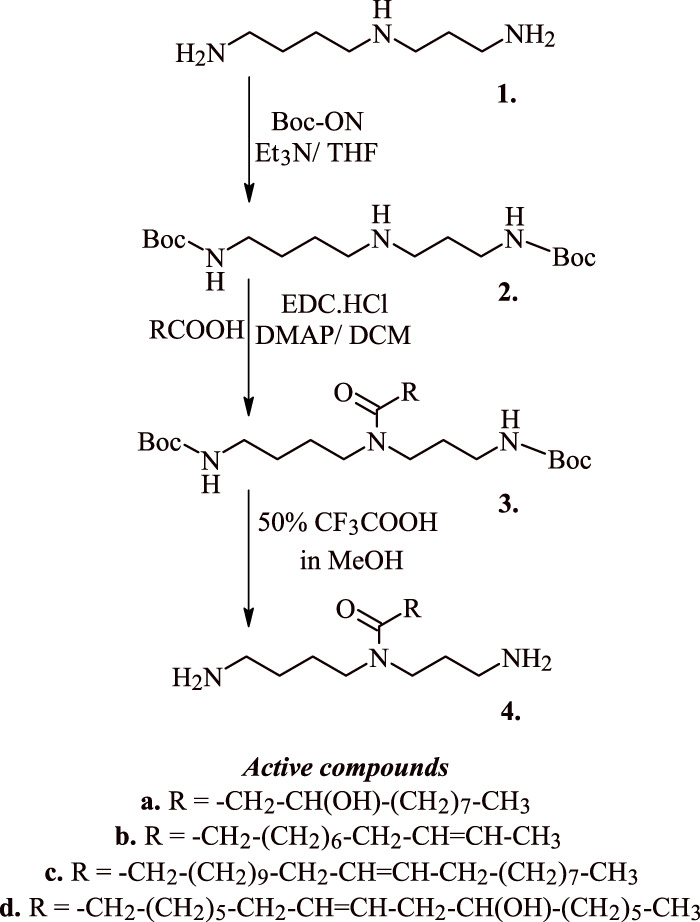
Synthesis of active acyl spermidine derivatives that induce apoptosis in cancer cells.

Polyamine analogues like spermidine have been reported to onset apoptotic cascades in the cancers of breast, liver, colon, skin, and the prostate. Razvi et al. (2017) have earlier reported the pro-apoptotic activity of acyl spermidine derivatives (**1–4** Figure 3) on human breast cancer cell lines and T-lymphoblastic leukemia cells. Reportedly, the most active compounds **4a-c (** Figure 3 **)** induced 12%–14% apoptosis in the Jurkat cells at concentration 50 μM, while compound **4d** (Figure 3) induced a significant 70% apoptosis under the same conditions. Exposure of Jurkat cells to acyl spermidine has shown a rapid surge in the function of annexin V positive cells. The onset of apoptosis is both time and dose dependent where the lipophilicity of acetyl spermidine plays a key role in regulating cytotoxicity towards tumor cells. Lipophilicity of this environment is due to the presence of alkyl side chain that enables its penetration through cytoplasmic membrane *via* endocytosis (Razvi et al., 2017).

**FIGURE 4:**
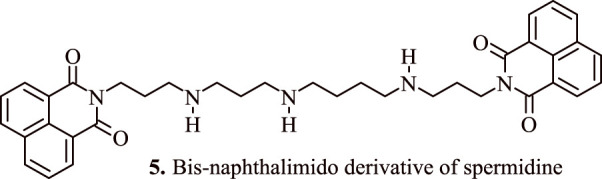
Bis-naphthalimido derivative of spermidine that indices apoptosis in adenocarcinoma cell lines.

The anticancer potential of DNA-intercalating Bis-naphthalimido compound (**5,** Figure 4) using a spermidine linker (BNIPSpd) has been tested by Ralton et al. (2009). A 48 h exposure of BNIPSpd indicated cytotoxicity towards HT-29 and Caco-2 colon adenocarcinoma cells with an IC50 value of 1.64 µM, and 0.15 µM. At 4 h, 0.5 µM BNIPSpd exposure caused a considerable damage to DNA while after 24 h, the expression of active caspase-3 was upregulated in a dose dependent manner. The appearance of chromatin condensation and fragmentation of internucleosomal DNA validated the onset of apoptosis, which is the main reason for the cytotoxicity in the test cancer cell lines (Ralton et al., 2009).

**FIGURE 5:**
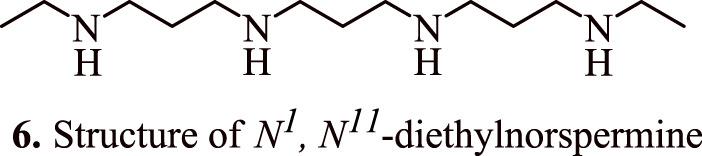
Structure of *N* ^*1*^ *, N* ^*11*^-diethylnorspermine that shows synergistic killing of colon carcinoma cells in combination with 5-fluorouracil.

Spermidine metabolism in colon carcinoma cells is also influenced by 5-fluorouracil (5-FU), a potent chemotherapeutic agent for HCT116 colorectal adenocarcinoma. A combination of 5-fluorouracil and spermine analogues *N* ^*1*^ *, N* ^*11*^-diethylnorspermine (DENSPM) (**6,** Figure 5) at concentrations 1.25, 2.5, 5, and 10 μM or α-difluoromethylornithine (DFMO) led to a synergistic killing of HCT116 colon carcinoma cells and upregulation in the transcript levels of the enzyme spermidine/spermine N1-acetyltransferase, which is involved in polyamine catabolism. The levels of spermine and spermidine were found to be depleted, while the level of acetylated spermidine increased which had led to tumour cell apoptosis in p53 null and p53 wild type variants. Interestingly, the combination therapy of DENSPM and 5-FU resulted in the activation of caspase 9. In addition, it also resulted in the suppression of cytochrome c oxidase, and NADH dehydrogenase, as evidenced by a surge in the levels of H_2_O_2_, and loss of membrane potential of mitochondria followed by a subsequent release of cytochrome c (Choi et al., 2005).

Amine oxidases catalyse the oxidative deamination of polyamines to generate peroxides and aldehydes. Ohkubo et al. (2019) reported the application of maize polyamine oxidase as a potential strategy towards anticancer therapy. The end products of enzyme catalysis of polyamines are considered responsible for its corresponding anticancer potential. The treatment of exogeneous spermine with maize polyamine oxidase caused a significant decline in the cell viability of multidrug-resistant colon adenocarcinoma (LoVo DX) in a time-dependent and dose-dependent manner. The flow cytometry analysis further indicated that the treatment of LoVo WT and LoVo DX cells with maize polyamine oxidase caused a considerable increase in their apoptotic population. The depolarization of mitochondrial membrane potential in LoVo DX cells declined remarkably on exposure to spermidine, which also determines apoptosis and cell viability (Ohkubo et al., 2019).

**FIGURE 6:**
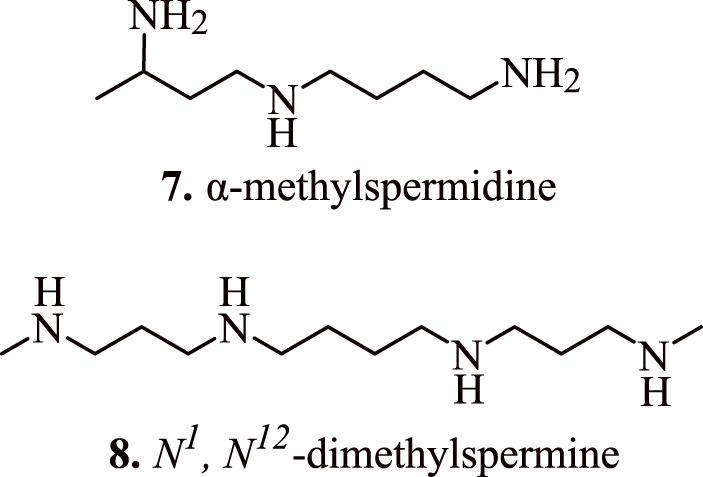
Structure of apoptosis inducing α-methylspermidine (α-MeSpd) and *N* ^*1*^ *, N* ^*12*^-dimethylspermine (Me2Spm).

The role of spermidine in the regulation of apoptosis was further established by Mandal et al. (2015) who transduced human embryonic kidney (HEK 293T) cells with adenovirus vector encoding spermidine N1-acetyltransferase 1 (AdSAT1) that caused a depletion of spermidine and spermine, restricted cell growth, eventually leading to a loss of cell viability. The investigations conducted *via* Annexin V/propidium iodide FACS, caspase assays, and analysis with terminal uridine nucleotide end-labelling (TUNEL) indicated obvious initiation of apoptosis in AdSAT1-transduced cells at 24–72 h. Induction of apoptosis in the spermidine-depleted cells is shown to occur *via* mitochondrial intrinsic pathway as validated by the deregulation of the membrane potential of mitochondria, downregulation of anti-apoptotic Bcl-xl, Mcl-1, and Bcl2. In addition, it also resulted in increased levels of pro-apoptotic Bax followed by the release of cytochrome c from mitochondria on transduction with AdSAT1 (Mandal et al., 2015). The appearance of cell shrinkage, nuclear fragmentation, vacuolization, membrane blebbing, and mitochondrial alteration indicate the onset of apoptosis. Furthermore, the generation of apoptosis due to the depletion of spermidine is established by the fact that polyamine analogues, namely, α-methylspermidine **(7,** Figure 6 **)** (α-MeSpd) and *N* ^*1*^ *, N* ^*12*^-dimethylspermine **(8,** Figure 6 **)** (Me2Spm), not the substrates of SAT1, partially restore the growth and inhibit apoptosis of AdSAT1-transduced cells. Similarly, the growth of AdSAT1-transduced cells isn’t restored by the inhibition of polyamine oxidases, which further indicated the non-involvement of accelerated polyamine metabolism for inducing growth arrest and apoptosis. These observations suggest the association of spermidine and polyamine analogues in the regulation of apoptosis (Mandal et al., 2015).

## Spermidine/spermine N1-acetyltransferase (SSAT)-induced anticancer effect

**FIGURE 7:**
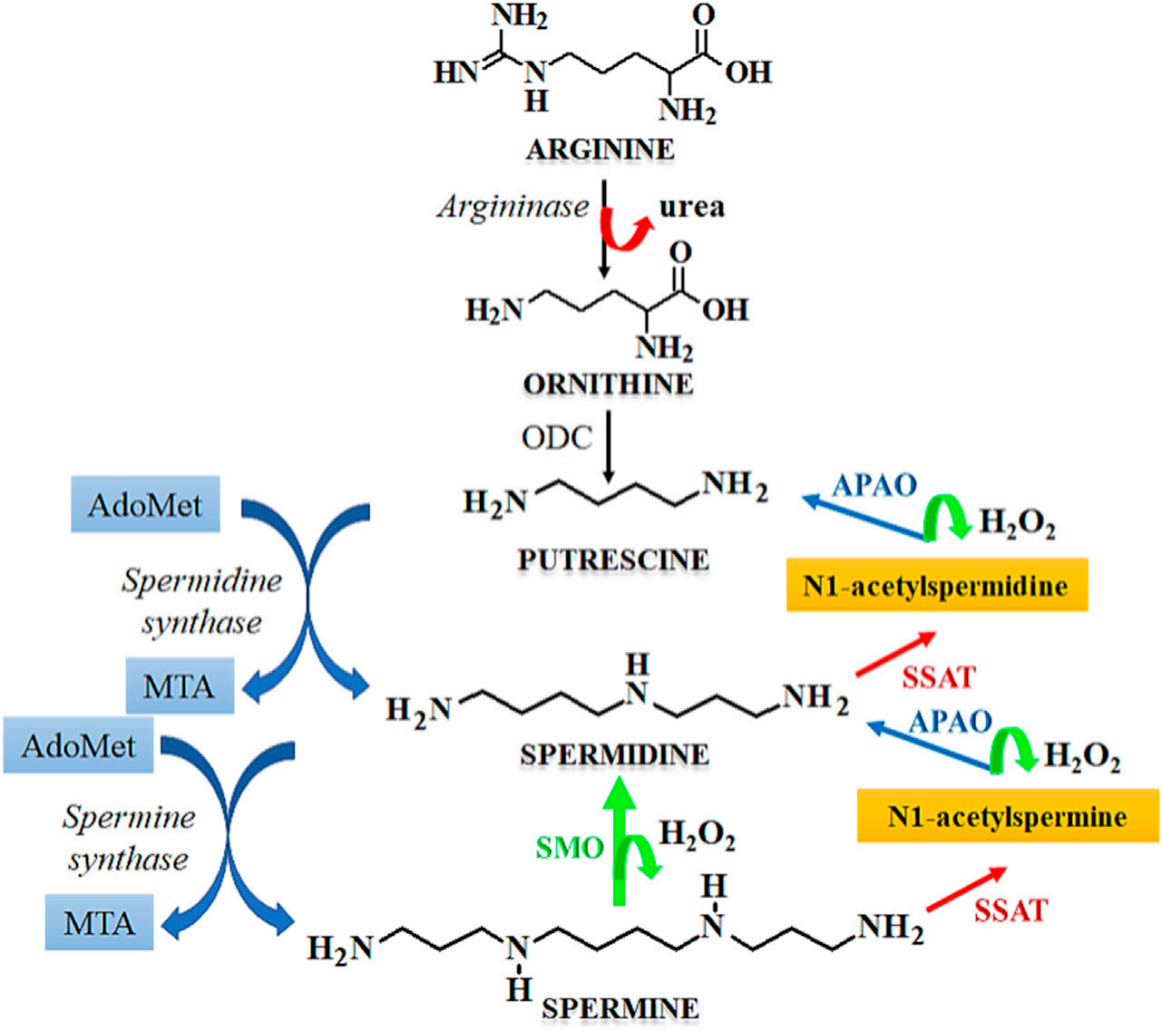
Mechanism of polyamine metabolism and the role of SSAT. Abbreviations: AdoMet, S-Adenosylmethionine; MTA, Metastasis associated protein; APAO, N1 acetylpolyamine oxidase; SSAT, Spermidine/spermine N1-acetyltransferase; ODC, Ornithine decarboxylase; SMO, smoothened receptor.

Cell cycle arrest is a desirable feature in the development of anticancer therapeutics as it leads to inhibition of the growth and progression of cancers. Spermidine in known to induce cell cycle arrest, which serves as a potential tool for the prevention of tumorigenesis. The metabolism of spermidine, and related polyamines such as spermine, putrescine is regulated by the expression of SSAT that maintains the polyamine homeostasis (Pegg, 2008). Figure 7 describes the role of SSAT in the metabolism of polyamines. Due to the above functions, the expression of SSAT enzyme serves as a diagnostic biomarker for human cancers (Maksymiuk et al., 2018). Under stressful physiological conditions, the expression of SSAT and its cellular concentrations are altered, which induces a cascade of events like cellular damage, oxidative stress, altered DNA dynamics, altered proliferation due to the dysregulation of cellular signaling pathways, and induction of cell cycle arrest. A heightened expression of SSAT has been observed in various malignancies due to which this enzyme holds a robust candidature for developing anticancer chemotherapeutics by serving as a target for substrate binding, whereas its metabolites may be used as novel cancer biomarkers. SSAT may also serve as a therapeutic target for rationally designed polyamine analogues that potentially modulate the enzyme expression thereby causing a surge in the cytotoxicity of cancer cells towards chemotherapy (Tse et al., 2022).

Cyclin A is necessary for entering the cell cycle at the S-phase, at which there is an increase in the levels of cyclin A. This is followed by its subsequent destruction before the beginning of metaphase. The cyclic dependent kinases (CDKs) that are associated with cyclin A play a central role in DNA replication and gaining entry into the S-phase by phosphorylation of the components associated with the DNA replication framework (Ding et al., 2020). The transcriptional factors, such as E2F are reported to govern the synthesis of cyclin A by their release from the pre-existing inactive complex and hypophosphorylated retinoblastoma. Similarly, E2F-1 is a primary upstream regulator of cyclin A which is activated in colorectal cancer cells (Fang et al., 2020) and its upregulation is found to be related with aggressive and malignant behavior of colorectal cancers. Sun et al. (2009) studied the effect of Spermidine/spermine N1-acetyltransferase (SSAT) on the cell cycle of colorectal cancer cells. SSAT expressing recombinant adenovirus, Ad-SSAT was reported to cause a surge in the enzyme expression, where it limited the growth of HT-29 cells by arresting the cell cycle in S-phase. As seen from the reporter gene assay, Ad-SSAT suppressed the expression of nuclear factor E2F-1 and cyclin A in Lovo cells and HT-29 cells (Sun et al., 2008a).

**FIGURE 8:**
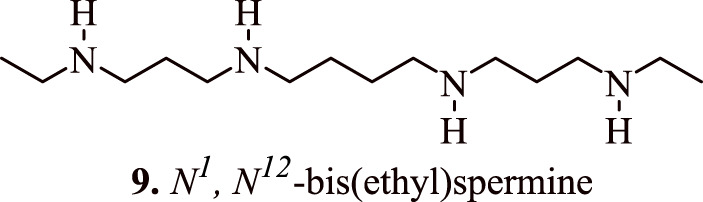
Structure of *N* ^*1*^ *, N* ^*12*^-bis(ethyl) spermine that inhibits the cancer cell growth by regulating the proliferation of cancer cells.

Several studies have confirmed the association between polyamine analogues and SSAT inhibitors with regulation/modulation of cancer cell proliferation. The polyamine analogue *N* ^*1*^ *, N* ^*12*^-bis(ethyl) spermine **(9,** Figure 8 **)** (BESPM) has been reported to inhibit cell growth in MALME-3 human melanoma cell lines by depleting the intracellular polyamine pools and by decreasing the levels of ornithine and S-adenosylmethionine decarboxylase (Bernacki et al., 1992). The administration of BESPM three times a day at 10, 20, and 40 mg/kg for 6 days completely suppressed the growth of established (100–200 mm^3^) MALME-3 tumour. Interestingly, BESPM-treated MALME-3 human melanoma cells exhibited an increased level of SSAT from 50 pmol/min/mg to more than 10,000 pmol/min/mg, and from 16 pmol/min/mg to 120 pmol/min/mg in LOX cells over 48 h due to an increased enzyme synthesis in these cell lines. The exposure of MALME-3 cells with BESPM caused an accumulation of N-acetylspermidine and an increased excretion of spermidine, putrescine, and N-acetylspermidine that led to polyamine pool depletion. Overall, it was inferenced that an increase in the SSAT activity serves as a determining factor in the growth sensitivity of target cells towards spermidine analogues, which further serves as a fascinating approach towards the designing of impending anticancer chemotherapeutics. In addition to a significant attenuation of tumour cell proliferation, the SSAT-mediated polyamine depletion also prevents their malignancy in Be17402 hepatocellular and HT-29 colorectal carcinoma cells. This effect is observed due to the suppression of the expression of GSK3β, and p-Akt, in addition to the nuclear translocation of β-catenin (Porter et al., 1991).

The role of SSAT in improving the sensitivity and responsiveness of cancer cells towards chemotherapeutics has been studied by Allen et al. (2007). SSAT is induced in response to 5-fluorouracil or oxaliplatin in the drug resistant HCT116 cell lines due to the upregulation of SSAT mRNA. This event is further potentiated in the presence of chemotherapeutic agents combined with polyamine analogue *N* ^*1*^ *, N* ^*11*^-diethylnorspermine (DENSpm) which are known to deplete polyamine pool by inducing SSAT. The reported combination therapy of 0.1, 1, or 10 μmol/L of DENSpm with 0.5, 5, or 10 μmol/L of oxaliplatin improved the cancer cell chemosensitivity, which was earlier resistant to the chemotherapeutic agents, mainly oxaliplatin. The loss of synergy between DENSpm and chemotherapeutics in response to the SiRNA-mediated downregulation of SSAT further confirms this observation. Table 1 indicates the role of modulated SSAT enzyme expression in the regulation of chemotherapy resistant/sensitive cancers (Allen et al., 2007).

**TABLE 1:** The role of modulated SSAT enzyme expression in the regulation of chemotherapy resistant/sensitive cancers.

SSAT expression modulating agent	Cancer cell lines	Effect	References
Bis(ethyl)polyamine analogues	Large cell undifferentiated lung carcinoma line NCI H157, and human small cell lung carcinoma cell line NCI H82	Increased sensitivity of cancer cells towards 2-difluoromethylornithine chemotherapy	Casero et al. (1989)
N1, N12-bis(ethyl)spermine	Cisplatin-sensitive and -resistant C13* ovarian cancer cells	Cisplatin-resistance causes a modulation of SSAT response towards BESpm at transcriptional and post-transcriptional levels	Marverti et al. (2001)
Lithium chloride	Ehrlich ascites tumour cells	LiCl treatment of Ehrlich ascites tumour cells increase the activity of SSAT during translation mediated by protein kinase C	Matsui-Yuasa et al. (1992)
Aspirin	Caco-2 colon cancer cells	Aspirin-induced SSAT causes depletion in cellular content of polyamines, leading to decreased carcinogenesis and chemopreventive actions in colorectal cancer.	Babbar et al. (2006)
Hydrogen peroxide or hyperoxia	Human breast cancer cell line MCF-7	Hydrogen peroxide produced 6-7-fold increase in total activity of SSAT, while Hyperoxia produced approximately a 3-fold increase in total SSAT activity eventually leading to tumour inhibition	Chopra and Wallace (1998)
Oxaliplatin, 5-fluorouracil and*N* ^*1*^ *, N* ^*11*^diethylnorspermine	Colorectal cancer cells HCT-116	Depletion of cellular spermine and spermidine pool due to higher levels of SSAT, thereby producing anticancer effect	Hector et al. (2008)
Indomethacin	Colon cancer cells HCT-116, and Caco-2	Increase in the SSAT activity, increased acetylation of polyamines and their efflux from colon cancer cells. These factors contribute towards impaired growth of cancer cells	Turchanowa et al. (2001)
Sulindac sulfone	Colon cancer Caco-2 cells	Induction of SSAT activity	Babbar et al. (2003)
Adenovirus	HT-29 and LoVo cells	Increased expression of SSAT	Sun et al. (2008b)
Polyamine Analogues N1-Ethyl-N11-((cyclopropyl)methyl)-4,8-diazaundecane and N1-Ethyl-N11-((cycloheptyl)methyl)-4,8- diazaundecane	three human prostate cancer cell lines, LNCaP, PC3, and Du145	Increased SSAT expression, depletion of polyamine pools	McCloskey et al. (2000)
Polyamine analog *N* ^*1*^ *, N* ^*11*^-diethylnorspermine	Four human prostate cancer cell lines, i.e., PC-3, TSU-pr1, DU-145, and JCA-1	Induction of SSAT expression	Schipper et al. (2000)
Combination of platinum drugs and *N* ^*1*^ *, N* ^*11*^-diethylnorspermine	A2780 human ovarian carcinoma cells and their oxaliplatin- and cisplatin-resistant variants A2780/C10B, and A2780/CP	Induction of SSAT mRNA	Tummala et al. (2011)
*N* ^*1*^ *, N* ^*11*^-diethylnorspermine	MCF-7 breast cancer cells	Increased spermidine metabolism by induction of SSAT, ROS generation, improved sensitivity of cancer cells towards Paclitaxel	Akyol et al. (2016)
*N* ^*1*^ *, N* ^*11*^-diethylnorspermine	Glioblastoma multiforme	Activation of SSAT expression	Jiang et al. (2007)
7β-hydroxycholesterol and 7β-hydroxysitosterol	Human colon cancer cells	Perturbation in SSAT expression	Roussi et al. (2006)
*N* ^*1*^ *, N* ^*11*^-Bis(ethyl)norspermine	Non-small cell lung carcinoma	Induction of SSAT expression	Gabrielson et al. (1999)

## Conclusion

The anticancer potential of spermidine is well established through various *in vivo* and *in vitro* studies. There is a large body of evidence that further supports the potential of spermidine in various ailments. Spermidine is also shown to have neuroprotective action, anti-ageing activity, and anti-inflammatory potency. The principal role of spermidine and related polyamines is its maintenance of physiologically optimal cell functioning and metabolism. Spermidine exerts anticancer effects by regulating cellular apoptosis, cellular autophagy, and by modulating or inducing the expression of enzymes such as monoamine oxidase (MAO) and SSAT. Spermidine analogues when administered with established anticancer chemotherapeutics provide a synergistic inhibition of tumour growth and related malignancies, such as colon cancer, hepatocellular carcinoma, breast cancer and prostate cancer. Increased spermidine intake is shown to maintain cellular homeostasis and also in the suppression of tumorigenesis. It is also known to support the growth of several established tumours. The enzymes involved in spermidine metabolism also play a key role in cancer management that form an attractive target for the development of anticancer chemotherapeutics. Furthermore, the clinical translation of spermidine and its analogues is still a subject of wide interest and needs materialization for the advancement of impending anticancer medications.

## References

[B1] Akyol Z. Coker-Gurkan A. Arisan E. D. Obakan-Yerlikaya P. Palavan-Unsal N. (2016). DENSpm overcame Bcl-2 mediated resistance against Paclitaxel treatment in MCF-7 breast cancer cells via activating polyamine catabolic machinery. Biomed. Pharmacother. 84, 2029–2041. 10.1016/j.biopha.2016.11.016 27881234

[B2] Allen W. L. Mclean E. G. Boyer J. Mcculla A. Wilson P. M. Coyle V. (2007). The role of spermidine/spermine N1-acetyltransferase in determining response to chemotherapeutic agents in colorectal cancer cells. Mol. Cancer Ther. 6, 128–137. 10.1158/1535-7163.mct-06-0303 17237273

[B3] Babbar N. Gerner E. W. Casero R. A. JR. (2006). Induction of spermidine/spermine N1-acetyltransferase (SSAT) by aspirin in Caco-2 colon cancer cells. Biochem. J. 394, 317–324. 10.1042/bj20051298 16262603 PMC1386030

[B4] Babbar N. Ignatenko N. A. Casero R. A. JR. Gerner E. W. (2003). Cyclooxygenase-independent induction of apoptosis by sulindac sulfone is mediated by polyamines in colon cancer. J. Biol. Chem. 278, 47762–47775. 10.1074/jbc.m307265200 14506281

[B5] Bernacki R. J. Bergeron R. J. Porter C. W. (1992). Antitumor activity of N,N'-bis(ethyl)spermine homologues against human MALME-3 melanoma xenografts. Cancer Res. 52, 2424–2430. 1568212

[B6] Casero R. A. JR. Celano P. Ervin S. J. Porter C. W. Bergeron R. J. Libby P. R. (1989). Differential induction of spermidine/spermine N1-acetyltransferase in human lung cancer cells by the bis(ethyl)polyamine analogues. Cancer Res. 49, 3829–3833. 2544259

[B7] Chen Y. Zhuang H. Chen X. Shi Z. Wang X. (2018). Spermidine-induced growth inhibition and apoptosis via autophagic activation in cervical cancer. Oncol. Rep. 39, 2845–2854. 10.3892/or.2018.6377 29693131

[B8] Choi W. Gerner E. W. Ramdas L. Dupart J. Carew J. Proctor L. (2005). Combination of 5-fluorouracil and N1,N11-diethylnorspermine markedly activates spermidine/spermine N1-acetyltransferase expression, depletes polyamines, and synergistically induces apoptosis in colon carcinoma cells. J. Biol. Chem. 280, 3295–3304. 10.1074/jbc.m409930200 15546879 PMC3584635

[B9] Chopra S. Wallace H. M. (1998). Induction of spermidine/spermine N1-acetyltransferase in human cancer cells in response to increased production of reactive oxygen species. Biochem. Pharmacol. 55, 1119–1123. 10.1016/s0006-2952(97)00601-1 9605436

[B10] Ding L. Cao J. Lin W. Chen H. Xiong X. Ao H. (2020). The roles of cyclin-dependent kinases in cell-cycle progression and therapeutic strategies in human breast cancer. Int. J. Mol. Sci. 21, 1960. 10.3390/ijms21061960 32183020 PMC7139603

[B11] Fan J. Feng Z. Chen N. (2020). Spermidine as a target for cancer therapy. Pharmacol. Res. 159, 104943. 10.1016/j.phrs.2020.104943 32461185

[B12] Fang Z. Lin M. Li C. Liu H. Gong C. (2020). A comprehensive review of the roles of E2F1 in colon cancer. Am. J. Cancer Res. 10, 757–768. 32266089 PMC7136928

[B13] Fulda S. (2015). Targeting apoptosis for anticancer therapy. Semin. Cancer Biol. 31, 84–88. 10.1016/j.semcancer.2014.05.002 24859747

[B14] Gabrielson E. W. Pegg A. E. Casero R. A. JR. (1999). The induction of spermidine/spermine N1-acetyltransferase (SSAT) is a common event in the response of human primary non-small cell lung carcinomas to exposure to the new antitumor polyamine analogue N1,N11-bis(ethyl)norspermine. Clin. Cancer Res. 5, 1638–1641. 10430062

[B15] Gogvadze V. Orrenius S. (2006). Mitochondrial regulation of apoptotic cell death. Chem. Biol. Interact. 163, 4–14. 10.1016/j.cbi.2006.04.010 16730343

[B16] Hector S. Tummala R. Kisiel N. D. Diegelman P. Vujcic S. Clark K. (2008). Polyamine catabolism in colorectal cancer cells following treatment with oxaliplatin, 5-fluorouracil and N1, N11 diethylnorspermine. Cancer Chemother. Pharmacol. 62, 517–527. 10.1007/s00280-007-0633-2 17987291

[B17] Hwangbo H. Kim D. H. Kim M. Y. Ji S. Y. Bang E. Hong S. H. (2023). Auranofin accelerates spermidine-induced apoptosis via reactive oxygen species generation and suppression of PI3K/Akt signaling pathway in hepatocellular carcinoma. Fish. Aquatic Sci. 26, 133–144. 10.47853/fas.2023.e11

[B18] Jiang R. Choi W. Khan A. Hess K. Gerner E. W. Casero R. A. JR. (2007). Activation of polyamine catabolism by N1,N11-diethylnorspermine leads to cell death in glioblastoma. Int. J. Oncol. 31, 431–440. 10.3892/ijo.31.2.431 17611701

[B19] Kim D. H. Kim J. H. Hwangbo H. Kim S. Y. Ji S. Y. Kim M. Y. (2021). Spermidine attenuates oxidative stress-induced apoptosis via blocking Ca(2+) overload in retinal pigment epithelial cells independently of ROS. Int. J. Mol. Sci. 22, 1361. 10.3390/ijms22031361 33572992 PMC7866386

[B20] Kim J. H. Hong S. B. Lee J. K. Han S. Roh K. H. Lee K. E. (2015). Insights into autophagosome maturation revealed by the structures of ATG5 with its interacting partners. Autophagy 11, 75–87. 10.4161/15548627.2014.984276 25484072 PMC4502675

[B21] Li W. Zou J. Yue F. Song K. Chen Q. Mckeehan W. L. (2016). Defects in MAP1S-mediated autophagy cause reduction in mouse lifespans especially when fibronectin is overexpressed. Aging Cell 15, 370–379. 10.1111/acel.12441 26750654 PMC4783353

[B22] Liu K. Zhao E. Ilyas G. Lalazar G. Lin Y. Haseeb M. (2015). Impaired macrophage autophagy increases the immune response in obese mice by promoting proinflammatory macrophage polarization. Autophagy 11, 271–284. 10.1080/15548627.2015.1009787 25650776 PMC4502775

[B23] Liu R. Cui J. Sun Y. Xu W. Wang Z. Wu M. (2021). Autophagy deficiency promotes M1 macrophage polarization to exacerbate acute liver injury via ATG5 repression during aging. Cell Death Discov. 7, 397. 10.1038/s41420-021-00797-2 34930917 PMC8688512

[B24] Liu R. Li X. Ma H. Yang Q. Shang Q. Song L. (2020). Spermidine endows macrophages anti-inflammatory properties by inducing mitochondrial superoxide-dependent AMPK activation, Hif-1α upregulation and autophagy. Free Radic. Biol. Med. 161, 339–350. 10.1016/j.freeradbiomed.2020.10.029 33122005

[B25] Madeo F. Bauer M. A. Carmona-Gutierrez D. Kroemer G. (2019). Spermidine: A physiological autophagy inducer acting as an anti-aging vitamin in humans? Autophagy 15, 165–168. 10.1080/15548627.2018.1530929 30306826 PMC6287690

[B26] Madeo F. Carmona-Gutierrez D. Kepp O. Kroemer G. (2018a). Spermidine delays aging in humans. Aging (Albany NY) 10, 2209–2211. 10.18632/aging.101517 30082504 PMC6128428

[B27] Madeo F. Eisenberg T. Pietrocola F. Kroemer G. (2018b). Spermidine in health and disease. Science 359, eaan2788. 10.1126/science.aan2788 29371440

[B28] Maksymiuk A. W. Sitar D. S. Ahmed R. Cheng B. Bach H. Bagchi R. A. (2018). Spermidine/spermine N1-acetyltransferase-1 as a diagnostic biomarker in human cancer. Future Sci. OA 4, FSO345. 10.4155/fsoa-2018-0077 30450232 PMC6234463

[B29] Mandal S. Mandal A. Park M. H. (2015). Depletion of the polyamines spermidine and spermine by overexpression of spermidine/spermine N(1)-acetyltransferase 1 (SAT1) leads to mitochondria-mediated apoptosis in mammalian cells. Biochem. J. 468, 435–447. 10.1042/bj20150168 25849284 PMC4550555

[B30] Marverti G. Bettuzzi S. Astancolle S. Pinna C. Monti M. G. Moruzzi M. S. (2001). Differential induction of spermidine/spermine N1-acetyltransferase activity in cisplatin-sensitive and -resistant ovarian cancer cells in response to N1,N12-bis(ethyl)spermine involves transcriptional and post-transcriptional regulation. Eur. J. Cancer 37, 281–289. 10.1016/s0959-8049(00)00389-0 11166157

[B31] Matsui-Yuasa I. Obayashi M. Hasuma T. Otani S. (1992). Enhancement of spermidine/spermine N1-acetyltransferase activity by treatment with lithium chloride in Ehrlich ascites tumor cells. Chem. Biol. Interact. 81, 233–242. 10.1016/0009-2797(92)90080-5 1311643

[B32] Mccloskey D. E. Woster P. M. Casero R. A. JR. Davidson N. E. (2000). Effects of the polyamine analogues N1-ethyl-N11-((cyclopropyl)methyl)-4,8-diazaundecane and N1-ethylN-11-((cycloheptyl)methyl)-4,8-diazaundecane in human prostate cancer cells. Clin. Cancer Res. 6, 17–23. 10656427

[B33] Nampoothiri M. Kolathur K. K. Sankhe R. Satarker S. (2023). “Spermidine, an autophagy inducer, as a therapeutic antiaging strategy,” in Emerging anti-aging strategies (Springer).

[B34] Ni Y. Q. Liu Y. S. (2021). New insights into the roles and mechanisms of spermidine in aging and age-related diseases. Aging Dis. 12, 1948–1963. 10.14336/ad.2021.0603 34881079 PMC8612618

[B35] Ohkubo S. Mancinelli R. Miglietta S. Cona A. Angelini R. Canettieri G. (2019). Maize polyamine oxidase in the presence of spermine/spermidine induces the apoptosis of LoVo human colon adenocarcinoma cells. Int. J. Oncol. 54, 2080–2094. 10.3892/ijo.2019.4780 31081059 PMC6521933

[B36] Pegg A. E. (2008). Spermidine/spermine-N(1)-acetyltransferase: A key metabolic regulator. Am. J. Physiol. Endocrinol. Metab. 294, E995–E1010. 10.1152/ajpendo.90217.2008 18349109

[B37] Pfeffer C. M. Singh A. T. K. (2018). Apoptosis: A target for anticancer therapy. Int. J. Mol. Sci. 19, 448. 10.3390/ijms19020448 29393886 PMC5855670

[B38] Pietrocola F. Castoldi F. Kepp O. Carmona-Gutierrez D. Madeo F. Kroemer G. (2019). Spermidine reduces cancer-related mortality in humans. Autophagy 15, 362–365. 10.1080/15548627.2018.1539592 30354939 PMC6333461

[B39] Pietrocola F. Lachkar S. Enot D. P. Niso-Santano M. Bravo-San Pedro J. M. Sica V. (2015). Spermidine induces autophagy by inhibiting the acetyltransferase EP300. Cell Death Differ. 22, 509–516. 10.1038/cdd.2014.215 25526088 PMC4326581

[B40] Pistritto G. Trisciuoglio D. Ceci C. Garufi A. D'Orazi G. (2016). Apoptosis as anticancer mechanism: Function and dysfunction of its modulators and targeted therapeutic strategies. Aging (Albany NY) 8, 603–619. 10.18632/aging.100934 27019364 PMC4925817

[B41] Porter C. W. Ganis B. Libby P. R. Bergeron R. J. (1991). Correlations between polyamine analogue-induced increases in spermidine/spermine N1-acetyltransferase activity, polyamine pool depletion, and growth inhibition in human melanoma cell lines. Cancer Res. 51, 3715–3720. 2065327

[B42] Ralton L. D. Bestwick C. S. Milne L. Duthie S. Kong Thoo Lin P. (2009). Bisnaphthalimidopropyl spermidine induces apoptosis within colon carcinoma cells. Chem. Biol. Interact. 177, 1–6. 10.1016/j.cbi.2008.09.033 18983836

[B43] Razvi S. S. Choudhry H. Moselhy S. S. Kumosani T. A. Hasan M. N. Zamzami M. A. (2017). Synthesis, screening and pro-apoptotic activity of novel acyl spermidine derivatives on human cancer cell lines. Biomed. Pharmacother. 93, 190–201. 10.1016/j.biopha.2017.06.019 28633130

[B44] Rider J. E. Hacker A. Mackintosh C. A. Pegg A. E. Woster P. M. Casero R. A. JR. (2007). Spermine and spermidine mediate protection against oxidative damage caused by hydrogen peroxide. Amino Acids 33, 231–240. 10.1007/s00726-007-0513-4 17396215

[B45] Roussi S. Gosse F. Aoude-Werner D. Zhang X. Geoffroy P. Miesch M. (2006). Perturbation of polyamine metabolism and its relation to cell death in human colon cancer cells treated by 7beta-hydroxycholesterol and 7beta-hydroxysitosterol. Int. J. Oncol. 29, 1549–1554. 17088995

[B46] Sacitharan P. K. Lwin S. Gharios G. B. Edwards J. R. (2018). Spermidine restores dysregulated autophagy and polyamine synthesis in aged and osteoarthritic chondrocytes via EP300. Exp. Mol. Med. 50, 1–10. 10.1038/s12276-018-0149-3 PMC6145946 30232322

[B47] Schipper R. G. Deli G. Deloyer P. Lange W. P. Schalken J. A. Verhofstad A. A. (2000). Antitumor activity of the polyamine analog N(1), N(11)-diethylnorspermine against human prostate carcinoma cells. Prostate 44, 313–321. 10.1002/1097-0045(20000901)44:4<313:aid-pros8>3.0.co;2-d 10951496

[B48] Sun H. Liu B. Wang W. Jiang G. S. Li W. Yang Y. P. (2008a). Adenovirus-mediated expression of spermidine/spermine N1-acetyltransferase gene induces S-phase arrest in human colorectal cancer cells. Oncol. Rep. 20, 1229–1235. 18949426

[B49] Sun H. Liu B. Yang Y. P. Xu C. X. Yan Y. F. Wang W. (2008b). Adenovirus-mediated expression of SSAT inhibits colorectal cancer cell growth *in vitro* . Acta Pharmacol. Sin. 29, 606–613. 10.1111/j.1745-7254.2008.00779.x 18430370

[B50] Sun Q. Fan W. Zhong Q. (2009). Regulation of Beclin 1 in autophagy. Autophagy 5, 713–716. 10.4161/auto.5.5.8524 19372752 PMC2789700

[B51] Tse R. T. Ding X. Wong C. Y. Cheng C. K. Chiu P. K. Ng C. F. (2022). The association between spermidine/spermine N(1)-acetyltransferase (SSAT) and human malignancies. Int. J. Mol. Sci. 23, 5926. 10.3390/ijms23115926 35682610 PMC9179984

[B52] Tummala R. Diegelman P. Hector S. Kramer D. L. Clark K. Zagst P. (2011). Combination effects of platinum drugs and N1, N11 diethylnorspermine on spermidine/spermine N1-acetyltransferase, polyamines and growth inhibition in A2780 human ovarian carcinoma cells and their oxaliplatin and cisplatin-resistant variants. Cancer Chemother. Pharmacol. 67, 401–414. 10.1007/s00280-010-1334-9 20443003 PMC3028085

[B53] Turchanowa L. Dauletbaev N. Milovic V. Stein J. (2001). Nonsteroidal anti-inflammatory drugs stimulate spermidine/spermine acetyltransferase and deplete polyamine content in colon cancer cells. Eur. J. Clin. Invest. 31, 887–893. 10.1046/j.1365-2362.2001.00901.x 11737227

[B54] Xu G. Jiang Y. Xiao Y. Liu X. D. Yue F. Li W. (2016). Fast clearance of lipid droplets through MAP1S-activated autophagy suppresses clear cell renal cell carcinomas and promotes patient survival. Oncotarget 7, 6255–6265. 10.18632/oncotarget.6669 26701856 PMC4868754

[B55] Yue F. Li W. Zou J. Jiang X. Xu G. Huang H. (2017). Spermidine prolongs lifespan and prevents liver fibrosis and hepatocellular carcinoma by activating MAP1S-mediated autophagy. Cancer Res. 77, 2938–2951. 10.1158/0008-5472.can-16-3462 28386016 PMC5489339

[B56] Yun C. W. Lee S. H. (2018). The roles of autophagy in cancer. Int. J. Mol. Sci. 19, 3466. 10.3390/ijms19113466 30400561 PMC6274804

